# The near-quantitative sampling of genomic DNA from various food-borne Eubacteria

**DOI:** 10.1186/s12866-014-0326-z

**Published:** 2014-12-31

**Authors:** Peter Irwin, Ly Nguyen, Yiping He, George Paoli, Andrew Gehring, Chin-Yi Chen

**Affiliations:** Agricultural Research Service, U. S. Department of Agriculture, Molecular Characterization of Foodborne Pathogens Research Unit, Eastern Regional Research Center, 600 East Mermaid Lane, Wyndmoor, PA 19038 USA

## Abstract

**Background:**

The disruption of the bacterial cell wall plays an important part in achieving quantitative extraction of DNA from Eubacteria essential for accurate analyses of genetic material recovered from environmental samples.

**Results:**

In this work we have tested a dozen commercial bacterial genomic DNA extraction methodologies on an average of 7.70 × 10^6^ (±9.05%), 4.77 × 10^8^ (±31.0%), and 5.93 × 10^8^ (±4.69%) colony forming units (CFU) associated with 3 cultures (*n* = 3) each of *Brochothrix thermosphacta* (*Bt*; Gram-positive), *Shigella sonnei* (*Ss*; Gram-negative), and *Escherichia coli* O79 (*Ec*; Gram-negative). We have utilized real-time PCR (*q*PCR) quantification with two specific sets of primers associated with the 16S *r*RNA “gene” to determine the number of copies CFU^-1^ by comparing the unknown target DNA *q*PCR results with standards for each primer set. Based upon statistical analyses of our results, we determined that the *Agencourt Genfind v2*, *High Pure PCR Template Prep Kit*, and *Omnilyse* methods consistently provided the best yield of genomic DNA ranging from 141 to 934, 8 to 21, and 16 to 27 16S *r*DNA copies CFU^-1^ for *Bt*, *Ss*, and *Ec*. If one assumes 6–7 copies of the 16S *r*RNA gene per genome, between 1 and 3 genomes per actively dividing cell and ≥ 100 cells CFU^-1^ for *Bt* (found to be a reasonable assumption using an optical method expounded upon herein) or between 1 and 2 cells CFU^-1^ for either *Ss* or *Ec*, then the *Omnilyse* procedure provided nearly quantitative extraction of genomic DNA from these isolates (934 ± 19.9 copies CFU^-1^ for *Bt*; 20.8 ± 2.68 copies CFU^-1^ for *Ss*; 26.9 ± 3.39 copies CFU^-1^ for *Ec*). The *Agencourt*, *High Pure*, and *Omnilyse* technologies were subsequently assessed using 5 additional Gram-positive and 10 Gram-negative foodborne isolates (*n* = 3) using a set of “universal” 16S *r*DNA primers.

**Conclusion:**

Overall, the most notable DNA extraction method was found to be the *Omnilyse* procedure which is a “bead blender” technology involving high frequency agitation in the presence of zirconium silicate beads.

**Electronic supplementary material:**

The online version of this article (doi:10.1186/s12866-014-0326-z) contains supplementary material, which is available to authorized users.

## Background

It is apparent that a quantitative understanding of microbial populations in various habitats can not be accomplished utilizing traditional culture methods [1] inasmuch as “unculturable” components may greatly outnumber all others [2,3]. The term unculturable is taken [4] to simply mean that current technology/methods do not permit the growth of the particular organism *in vitro* yet these same organisms may thrive, or at least survive, in their native habitat. Obviously organisms which are moribund or injured are also likely to be unculturable. Even when the organisms in question can be propagated, they are liable to be analytically under-represented using most plate counting methods [5]. One way to enumerate microbial populations *ex colliquiā* (literally, from [out of] the drain [or gutter]) is by means of a quantitative metagenomic analysis (*i.e.*, a sequence analysis of *all* the genetic material sampled from the “environment” for identification purposes) [6] given that the metagenome includes all organisms: culturable, viable but not culturable [4,7,8], as well as moribund/dead cells.

The disruption of the cell wall envelope [9] is one of the most important aspects of quantitative metagenomic analysis since the total isolation of DNA from all Eubacteria is required. These “true bacteria” consist of cells bounded by a cytoplasmic lipid membrane and either a thick peptidoglycan, also known as murein [10], layer (Gram-positive bacteria) or a thin peptidoglycan stratum layered between an inner and outer membrane (Gram-negative bacteria). This outer membrane contains lipopolysaccharides (LPS) which are made up of lipid A (the inner-most of three regions, typically a phosphorylated glucosamine disaccharide with multiple fatty acid side-chains), core polysaccharides (inner and outer core), and an O-antigen (3- to 8-mer oligosaccharides) [11].

There are numerous methods available for cell wall disruption including physical approaches (*e.g.*, particle-based disruption, sonication) [9,12-15], biochemical-based methods (*e.g.*, detergents, enzymes, *etc.*) [13,16-21] or some combination of the two [18,22]. Quantitative extraction of DNA is particularly difficult for some bacteria, especially the Gram-positives, which seem to be somewhat more resistant to cell wall dissolution. The “prototypal” [13] cell disruption method was published in 1961 [23] and used an enzyme/detergent extraction and organic solvents to remove low molecular weight solutes whereupon recovery of the DNA from an aqueous solution was made using alcohol precipitation. For chemical disruption, different compounds have been used to dissolve and/or disrupt the bacterial cell wall: enzymes such as lysozyme and proteinase K, detergents (*e.g.*, SDS), as well as other chaotropic compounds (*e.g.*, guanadinium isothiocyanate, phenol, urea) and chelating agents (*e.g.*, EDTA). Physical techniques are often the first method of choice for cell disruption, and include mechanical disruption, liquid homogenization, sonication, freeze-thawing, and manual grinding. Regardless of what protocol one uses, the DNA which is extracted should be in a form that can be employed for immediate analysis [19].

Our research project’s long-term goal is to quantitatively assess the relative composition of culturable [1] as well as all other forms of Eubacteria associated with meat processing, which can have as many as 10^5^ colony forming units (CFU) cm^-2^ [24] of area tested. Ultimately, we seek to determine if background organisms from such environments outgrow (at refrigeration temperatures) [1] and mask entrapped pathogens (*e.g.*, in biofilms or other structures) from detection in processed foods. Upon reviewing recent literature [9,12,13,15-22], it was unclear which, if any, commercial cell lysis/DNA extraction kits extract genomic DNA quantitatively (*i.e.*, with a near 100% efficiency). This latter statement is true because the subject tests were performed using a small, somewhat arbitrary, collection of techniques with little concern for statistical analyses of target DNA concentration. Additionally, primer-specific standards were not typically used to convert raw *q*PCR data to the total number of gene copies as a function of some standard cell concentration determinant such as most probable number (MPN), CFU or even microscopic cell counts. Thus, there is a need to investigate not only some recent next generation genomic DNA extraction procedures but also to do so using a more rigorous experimental design: *i.e.*, true replication on the major source of microbiological variability using at least 3 clonal isolates derived from various food products and CFU-normalized target DNA concentration (*i.e.*, DNA copies per CFU).

## Methods

### Definitions

#### Indices

- *i* = dilution index reserved for standard (*stnd*) 16S *r*DNA solutions used in *q*PCR (*i* = 0, 1, 2, ⋯, 6 ); *e.g.*, 0.1^*i=*3^ = 0.001 representing the dilution factor for three 1:10 dilutions of the *stnd* 16S *r*DNA sample (see [*T*]_*i* = 0_ below)
- *j* = dilution index reserved for unknown (*unk*) concentrations of genomic DNA from various extracts (*j* = 0, 1, 2, 3); *e.g.*, 0.1^*j=*0^ = 1 represents the undiluted sample; dilutions were performed from extracts in order to determine if raw *q*PCR efficiency data were within acceptable limits
- *k* = treatment (*e.g.*, extraction method) index (*k* = 1, 2, ⋯, *m*)
- *ℓ* = replicate or block index (*ℓ* = 1, 2, ⋯, *n*; typically *n* = 3 for all analysis of variance and multiple range tests, otherwise *n* is variable)
- *x*_*kl*_ = the *k*^th^ treatment (*e.g.*, extraction or isolate) and *ℓ*^th^ replicate (*e.g.*, block) of *any* set of experimental observations

#### Abbreviations, symbols, & equations

- *Bt* = *Brochothrix thermosphacta* (ground chicken isolate) [1]
- *Ss* = *Shigella sonnei* (ground chicken isolate) [1]
- *Ec* = *Escherichia coli* O79 (whole chicken carcass isolate; O-type determined [2 July 2013] by the *E. coli* Reference Center, The Pennsylvania State University, University Park, PA 16802)
- *R*_*n*_ = normalized fluorescence signal with respect to cycle number (*C* ) which is typically sigmoidal in shape (*i.e.*, ∂*R*_*n*_/∂*C* has a near-Gaussian line-shape)
- *C*_∂i or j_ = extrapolated cycle number where ∂^2^*R*_*n*_/∂*C*^2^ = 0 for any *i*^th^ or *j*^th^ dilution
- [*T*]_*i*_ = *i*^th^ dilution of the standard target gene (copies μL^−1^) solution being amplified; [*T*]_*i* = 0_ = 1.31 × 10^9^ 16S *r*DNA copies μL^−1^ (*Bt* standard used for all Gram-positive organisms), 1.06 × 10^9^ copies μL^−1^ (*Ss* standard used for all Gram-negative organisms except *Ec*), or 9.66 × 10^8^ copies μL^−1^ (*Ec* standard)
- $\frac{\partial {C}_{\partial i}}{\partial Lo{g}_{10}{\left[T\;\right]}_i}$ = change in *C*_∂i_ with *Log*_10_[*T*]_*i*_ (ideally ∂*C*_∂i_/∂*Log*_β_[*T*]_*i*_ = −*Log*_2_*β*; ∂*C*_∂i_/∂*Log*_*β*_[*T*]_*i*_ is always equivalent to ∂*C*_∂i_/∂*Log*_*β*_[*ϕ*^*i*^] and *ϕ* is the dilution factor; in this work *ϕ* = *β*^−1^ and *β*, the base of the logarithm, is always 10) [25]
- $\frac{\partial {C}_{\partial j}}{\partial Lo{g}_{10}\left[{0.1}^{\;j}\right]}$ = slope of *C*_∂*j*_ with respect to *Log*_10_[0.1^*j*^] (*i.e.*, dilutions of a DNA extract of unknown concentration; *j* = 0, 1, 2, or 3)
- *C*_∂int*obs*_ = *intercept* calculated from linear regression analysis of *C*_∂*i*_ as affected by changes in *Log*_10_[*T*]_*i*_ (ideally *C*_∂int*obs*_ = *C*_∂i_ + *Log*_2_[*T*]_*i*_) [25]
- *C*_∂int*predicted j*_= ${C}_{\partial j}-\left(\frac{\partial {C}_{\partial j}}{\partial Lo{g}_{10}\left[{0.1}^j\right]}\times Lo{g}_{10}\left[{\left(1+{\varepsilon}_i\right)}^{\left({C}_{\partial \operatorname{int}\;obs}-{C}_{\partial j}\right)}\right]\right)$; predicted *intercept* for each *j*^th^*unk* dilution; derivation of *C*_∂int*predicted j*_was fully developed elsewhere [25]
- ${\overline{C}}_{\partial \operatorname{int}\; predicted}$ = *C*_∂int*predicted j*_ averaged across all *j*
- *ε*_*stnd*_ = *Taq* DNA polymerase efficiency associated with standard dilutions = $-1+{10}^{{\left(-\partial {C}_{\partial i}/\partial Lo{g}_{10}{\left[T\;\right]}_{\kern0.1em i}\right)}^{-1}}$
- *ε*_*unk*_ = *Taq* DNA polymerase efficiency associated with unknown dilutions = $-1+{10}^{{\left(-\partial {C}_{\partial j}/\partial Lo{g}_{10}\left[{\phi}^{\;j}\right]\right)}^{-1}}$; poor *ε*_*unk*_ s (*e.g.*, 0.9 ≥ *ε*_*unk*_ ≥ 1.1) are possible indicators of enzyme perturbation [26] by inhibitory substances in an extract
- [*Ŧ*]_*j*_ = traditional calculation of the unknown target gene DNA concentration (copies μL^−1^ of extract) for the *j*^*th*^ dilution = ${\left(1+{\varepsilon}_{stnd}\right)}^{C_{\partial \operatorname{int}obs}\hbox{--}\ {C}_{\partial \kern0.37em j}}$
- [*T*]_*j*_ = *j*^th^ dilution of the corrected unknown DNA concentration (copies μL^−1^ of extract) = ${\left(1+{\varepsilon}_{unk}\right)}^{{\overline{C}}_{\partial \operatorname{int}\; predicted}\hbox{--}\ {C}_{\partial \kern0.37em j}}$; this calculation corrects [25] for the fact that *ε*_*stnd*_ sometimes is substantially different than *ε*_*unk*_ and is the value reported in all Tables; when *ε*_*stnd*_ ~ *ε*_*unk*_, [*Ŧ*]_*j*_ ~ [*T*]_*j*_
- *δ* = organism concentration or density (CFU mL^−1^)
- *r*RNA “gene” copies CFU^−1^ = [*T*]_*j*=0_ (in units of copies μL^−1^ of extract) × total μL of extract ÷ CFUs in 1 mL of culture; the values of the total assay volume have been provided at the end of each extraction procedure (listed below). The average value ($\overline{x}$) of each biological replicate’s CFU mL^-1^ are listed in all Tables ± coefficients of variation (*CV* = $s\div \overline{x}$). Since all counting-based data have technical replicate variances ~ $\overline{x}$ (assuming the number of observations/dilution were appropriately high), we report $\overline{x}$ and *CV* of the CFU mL^-1^ calculated from 2 or three 1:10 dilutions of the starting concentration.
- *EE* = extraction efficiency = observed 16S *r*DNA copies CFU^-1^ ÷ (16S *r*RNA gene copies genome^-1^ × genomes cell^-1^ × cells CFU^-1^ ÷ plating efficiency); *e.g.*, assuming 24 copies CFU^-1^ ÷ (7 copies genome^-1^ × 1.5 genomes cell^-1^ × 1.5 cells CFU^-1^ ÷ 0.67 plating efficiency [67%]), would result in a near 100% efficiency; gene copies genome^-1^ can vary between 1 and 14 but typically between 5 and 7; genomes cell^-1^ would probably vary between 1 and 3 but is dependent upon the rate of cell division; cells CFU^-1^ varies greatly depending on the organism but typically ranges between 1 and 2 for *Ss*, *Ec*, and *Salmonella* spp.; plating efficiency is a correction for losses on solid media (*e.g.*, for organisms like *Ss* and *Ec*, this term could vary between 50 and 100%)
- SS = sum of squares
- *TMS* = treatment mean square
- “=(Treatment SS/(*m*-1) ”
- *EMS* = error mean square
- “=(Total SS – (Block SS + Treatment SS))/((*m*-1)_*_(*n*-1))”
- *F* = F statistic
- “=*TMS*/*EMS* ”
- $$ {\overline{x}}_k={x}_{k \bullet}\div n $$
- “=AVERAGE(*x*_*k*1_׃*x*_*kn*_)”
- *SE* = experimental standard error
- “=(SQRT(*EMS*)/*n*)”
- *P* = the probability of rejecting the *null hypothesis* when it is true (*i.e.*, when there is no relationship between two measured observations); means were characteristically taken to be “significantly different” when *P* ≤ 0.05
- *q*_*P*_ = the “Studentized” range distribution tabulated in numerous statistics texts [27,28] for a *P* = 0.01 or 0.05
- *t*_*P*_ = “Student’s *t* ” at probability *P*
- “ =TINV(*P*, *n*-2)”

### Statistical analyses

The covariance (${\sigma}_{\eta_{\;1}\cdot {\eta}_{\;2}}^{\;2}$) associated with an hypothetical set of variables *η*_1,*l*_ and *η*_2,*l*_ (each with *n* replicates; *e.g.*, ∂*C*_∂i_/∂*Log*_10_[*T*]_*i*_, *C*_∂*j*_, or [*T*]_*i*_) was calculated using the Excel function “ =COVARIANCE.P(*η*_1,1_׃*η*_1,*n*_,*η*_2,1_׃*η*_2,*n*_)” and the 2 variances, ${\sigma}_{\eta_{\;1}}^{\;2}$ and ${\sigma}_{\eta_{\;2}}^{\;2}$, were calculated using “ =(STDEV.P(*η*_1,1_׃*η*_1,*n*_))^2” and “ =(STDEV.P(*η*_2,1_׃*η*_2,*n*_))^2”. Identical results were obtained using the Variance-Covariance matrix (2 × 2) approach whereupon ${\sigma}_{\eta_{\;1}\cdot {\eta}_{\;2}}^{\;2}$ is equal to the two off-diagonal terms and ${\sigma}_{\eta_{\;1}}^{\;2}$ or ${\sigma}_{\eta_{\;2}}^{\;2}$ are the diagonal components. The statistical relevance of ${\sigma}_{\eta_{\;1}\cdot {\eta}_{\;2}}^{\;2}$ was calculated by testing the significance of ${\rho}_{\eta_{\;1}\cdot {\eta}_{\;2}}$ = ${\sigma}_{\eta_{\;1}\cdot {\eta}_{\;2}}^{\;2}$ × $\sqrt[-2\;]{\sigma_{\eta_{\;1}}^{\;2}\cdot {\sigma}_{\eta_{\;2}}^{\;2}}$ (*i.e.*, the correlation coefficient) using a *t*-test (*e.g.*, *t*_*ρ*_ = $\left|{\rho}_{\eta_{\;1}\cdot {\eta}_{\;2}}\right|$ × $\sqrt{\frac{n-2}{1-{\rho}_{\eta_{\;1}\cdot {\eta}_{\;2}}^{\;2}}}$) at some level of probability *P*. Determination of this probability-level was made by continuously changing (using Excel’s *Solver* tool) this term in the Excel equation for *t*_*Ρ*_ until the TINV function matched that of *t*_*ρ*_. Such tests can be important for some comparisons since they determine the statistical significance of a correlation of paired variables which are not necessarily a direct function of one another but which might vary together (*i.e.*, non-randomly) because of subtle factors in the system being examined. All $\overline{x}$ values reported in figures/tables are presented ± (*n*-1)-weighted standard deviations (*s*; “ =STDEV.S(*x*_1,1_׃*x*_1,*n*_)”). One-way analyses of variance (ANOVA; always for *n* = 3) operations were performed assuming a randomized complete block design [27] and means were separated based upon a “Tukey Multiple Range” analysis which is also known as the “Honestly Significant Difference” (*HSD*) Test [28]. All ANOVA calculations and our algorithm for *HSD* are provided in the (Additional file 1 and Additional file 2).

### 16S *r*DNA standard solutions

One Gram-positive (*Bt*) and two species of Gram-negative (*Ss* and *Ec*) bacteria were streaked onto Luria-Bertani (LB; Difco, Detroit, MI, USA; 2% [w/v] agar) plates and grown overnight at room temperature whereupon a single colony of each was selected. All these strains had previously been isolated from commercially available ground or whole chicken and identified based upon 16S *r*RNA gene sequencing [1]. Each selected colony was mixed with 50 μL *PrepMan Ultra* (Applied Biosystems, Foster City, CA, USA), heated 15 min at 99°C in a thermocycler (*i*Cycler, BioRad, Hercules, CA, USA), cell debris was collected into a pellet by centrifugation (Eppendorf 5415R, Hamburg, Germany throughout), and the supernatant collected into a fresh tube. Amplification of the 16S *r*RNA gene was then performed as follows. Each PCR cocktail contained 25 μL GoTaq Green 2× (Promega, Madison, WI, USA), 5 μL (10 μM) each of EubA and EubB [29] forward and reverse primers, 14 μL PCR water (*i.e.*, free of all DNA, RNase, and DNase; Qiagen Sciences, Germantown, MD, USA), and 1 μL of the aforementioned genomic DNA template. Thermocycler conditions were as follows: DNA denaturation at 95°C for 90 s, 40 total cycles consisting of denaturing at 95°C for 30 s, annealing at 55°C for 45 s, extension at 72°C for 60 s [25]; as a final extension, samples were maintained at 72°C for 5 min.

Upon determining the presence of the 16S *r*RNA gene using gel electrophoresis (*ca*. 1400 bp product), the PCR products were purified using *AmPure* magnetic beads (Agencourt Bioscience [Beckman Coulter Inc.], Beverly, MA, USA) as detailed previously [1]. The concentration of these various *target* DNA standards ([*T*]_*i*_) was determined using a NanoDrop ND-1000 UV–VIS Spectrophotometer (NanoDrop Technologies Inc, Wilmington, DE, USA) where 2 μL of undiluted, purified PCR product was placed onto the apparatus and the OD measured (260 nm). OD_260_ values were converted to concentration (ng DNA μL^−1^) by comparing them with solutions of known DNA concentration (~67, 50, 38, 28, 21, 16, 12, and 0 ng μL^−1^ of Lambda DNA *HindIII* digest, Sigma-Aldrich, St. Louis, USA). Dilutions were made on this original cleaned-up standard so that the final concentration of DNA was ~ 10^9^ 16S *r*DNA copies per μL (*i.e.*, this defines [*T*]_*i* = 0_). When used as a *q*PCR standard set of solutions 5 additional 1:10 dilutions were made (*i.e.*, [*T*]_*i* = 1_ to [*T*]_*i* = 5_).

### *q*PCR

Sheared salmon sperm DNA (Ambion, Austin, TX, USA) was used to suppress the apparent binding of standard or unknown target DNA to the walls of the mixing tubes (RNase/DNase/pyrogen-safe Denville Scientific, Posi-Click, 1.7 mL polypropylene micro-centrifuge tubes). The salmon sperm DNA was diluted so that the final concentration was 4 ng per reaction. *QuantiFast* SYBR Green (Qiagen Sciences) was utilized where each polymerase chain reaction contained: 12.5 μL 2× *QuantiFast* SYBR green, 2.5 μL of a 10 μM stock solution of each forward and reverse primers (all primers herein are reported [5′ → 3′]; *Brochothrix*: Forward [Broc PA] = CAC AGC TGG GGA TAA CAT CGA, Reverse [Broc PB] = GGT CAG ACT TTC GTC CAT TGC C, 262 bp product; *Shigella*: Forward [Shig 2A] = TTA GCT CCG GAA GCC ACG, Reverse [Shig 2B] = ATA CTG GCA AGC TTG AGT CTC GT, 226 bp product), 6.5 μL PCR H_2_O (containing the 4 ng sheared salmon sperm DNA as a blocking reagent), and 1 μL template DNA (*i.e.*, either [*T*]_*i*_ or [*T*]_*j*_; *i* = 0, 1, ⋯ , 6 and *j* = 0, 1, 2, 3). All experiments were run on an Applied Biosystems 7500 FAST (Carlsbad, CA, USA) real-time PCR and the conditions were programmed according to the *QuantiFast* SYBR green protocol (Qiagen Sciences) [25]. DNA melt-curve [30] data were always collected and checked to confirm that the appropriate PCR product was being amplified in unknown DNA extractions. For testing isolates (see below) other than the above, a set of “universal” 16S *r*DNA primers was generated based upon minor modifications of oligonucleotides published by Nadkarni *et al*. [31]: Universal Forward (16S FU) = GTG CCA GCA GCC GCG GTA ATA C, Universal Reverse (16S RU) = GAC TAC CAG GGT ATC TAA TCC, 291 bp product. The aforementioned *Bt* standard was used for all Gram-positive and the *Ss* standard was utilized for all Gram-negative species. Using either set of standards resulted in approximately the same values for [*T*]_*j*_.

### *q*PCR data analysis

Derivative-based methods have been shown [32] to have an advantage over the threshold cycle number (*C*_*t*_) method in calculating *q*PCR results given that they require no baseline correction. In our usage, *C*_∂_ is the calculated cycle (*C* ) number where ∂^2^*R*_*n*_/∂*C*^2^ = 0 (*i.e.*, the maximum in ∂*R*_*n*_/∂*C*) which is based upon linear extrapolation from the two ∂^2^*R*_*n*_/∂*C*^2^ data points bounding 0 and constitute the last positive and first negative data points within the ∂^2^*R*_*n*_/∂*C*^2^ data. We have found (based on all the standards run herein) that there were only small differences between the *C*_∂_ and *C*_*t*_ method from the standpoint of either *C*_∂_ or *C*_*t*_ as a function of *Log*_10_[*T*]_*i*_ slopes: Method **1** = ∂*C*_*∂i*_/∂*Log*_10_[*T*]_*i*_ = −3.34 ± 0.0313 (*ε*_*stnd*_ = 0.961 ± 0.0125) and Method **2** = ∂*C*_*ti*_/∂*Log*_10_[*T*]_*i*_ = −3.42 ± 0.0214 (*ε*_*stnd*_ = 0.997 ± 0.0126). We also found that there was a highly significant covariance statistic (${\sigma}_{\mathbf{1}\cdot \mathbf{2}}^{\;2}$; *n* = 8 technical replicates × 3 isolates = 24 pairs of slopes) associated with the two methods alluded to above (*e.g.*, *ρ*_1⋅2_ = 0.623 [*t*_*ρ*_ = 3.74, *P* = 0.00114]) indicating that the two methods’ slopes vary significantly together which is reasonable, and expected, for different calculation methods used on the same “raw” data (*R*_*n*_ -*vs*- *C*). We prefer the *C*_∂_-based technique because it is better-defined and more objective than *C*_*t*_-based values which use thresholds assigned by thermocycler software (or user-defined). Most importantly, however, we have observed consistently smaller standard deviations (the average *s* across all observations was 0.304 for *C*_∂*i*_ and 0.522 for*C*_*ti*_) using the derivative method. All target DNA concentrations reported in Tables are based upon the [*T*]_*j*_ calculation provided in “Abbreviations, Symbols, & Equations” above.

### Organisms used for extraction method testing

#### Screening all extraction methods

*Bt*, *Ss* and *Ec* bacterial strains were streaked onto LB plates and incubated at 30°C until colonies were about 1 mm in size. Three separate colonies (clones) for each isolate were chosen and inoculated into 3 lots (one colony per lot) each of LB broth and grown at 30°C at 200 rpm (*i.e.*, *n* = 3; a randomized complete block design) [27]. After 16 hrs, each isolate’s 3 biological replicates were chilled in an ice-bath and 6 × 6 drop-plated [33] on solid LB media so that all DNA quantification could be eventually normalized to colony forming units (CFU). After plating, each overnight culture was broken up into numerous 1 mL aliquots, centrifuged at 13,000 rpm (15,682 × *g*), and supernatant discarded. These bacterial pellets were stored at -20°C until needed for each set of extractions. Immediately before use, pellets were thawed and re-centrifuged to remove excess liquid and/or condensation.

#### Quantitative performance of High Pure, Agencourt and Omnilyse DNA extraction protocols

Various bacterial strains (Gram-positive: Staphylococcus aureus* [RN4220; source: bovine mastitis]*, Streptococcus pneumoniae* [ground chicken]*, Enterococcus faecalis* [whole chicken]*, Lactococcus lactis* [salad bar lettuce]*, and Carnobacterium maltaromaticum* [pork sausage]; Gram-negative: *Salmonella* Typhi* [G8430, CDC]*, Pseudomonas oleovorans* [ground chicken]*, Aeromonas salmonicida* [raw shrimp]*, Kluyvera ascorbata* [salad bar lettuce]*, Pantoea agglomerans* [salad bar lettuce]*, Rahnella aquatilis* [salad bar lettuce & tomato]*, Acinetobacter lwoffii* [ground chicken]*, Hafnia alvei* [salad bar lettuce]*, Citrobacter frenundii* [ground chicken]*, Serratia proteamaculans* [ground chicken]), were streaked onto LB plates and incubated at 30°C until colonies were about 1 mm in size. The preceding bacterial isolates marked with an *asterisk* were obtained from a local microbiological collection and 4 primer-based 16S *r*DNA sequencing [1] was used to substantiate their *putative* identity. All other isolates were obtained from a collection produced during a population (culturable) study from this group previously published [1]. As before, 3 colonies (*i.e.*, *n* = 3 biological replicates) were chosen for each isolate and inoculated into LB broth at 30°C at 200 rpm. One isolate, *Pseudomonas oleovorans*, was also grown in tryptic soy broth/plates (TSB) due to our concern that this organism did not seem to grow as well on LB. Numerous 1 mL samples from the overnight culture were selected and centrifuged at 13,000 rpm and the supernatant was discarded. These bacterial pellets were then stored at -20°C until needed for the various extraction experiments. As previously mentioned, each of the isolates’ three cultures were also drop-plate enumerated so that all DNA quantification could be normalized to CFU.

### Extraction procedures and yields per mL of culture

#### Fast ID kit (Genetic ID NA, Inc, Fairfield, IA)

This genomic DNA extraction kit has been used [34] for isolating “high quality” DNA from eukaryotes such as higher plants. To each thawed bacterial pellet, 1 mL of Genomic Lysing buffer and 10 μL of Proteinase K solution (10 mg mL^-1^) were added. Samples were transferred to 2 mL tubes for easier handling and incubated at 65°C for 30 minutes in a water bath (Thermo Scientific 280 Series). Samples were centrifuged at 10,000 rpm (*i.e.*, 9,279 × *g*) for 5 minutes and 500 μL of the supernatant was transferred to another sterile 2 mL tube to which 500 μL of Genomic Bind buffer was added and mixed by pipetting. Another centrifugation at 10,000 rpm for 5 minutes followed and the supernatant was then pipetted into DNA columns (provided in kit). Columns were spun at 1,000 rpm (*i.e.*, 93 × *g*) for 5 minutes and the flow-through was discarded. The column membrane was washed once with 800 μL Genomic Wash Buffer and subsequently washed 3× with 800 μL of 75% EtOH making sure to discard the flow-through after each wash. After the last wash, the column was centrifuged briefly at high speed to completely dry the membrane. The column was then transferred to a sterile 1.7 mL centrifuge tube whereupon 100 μL of 1× Tris-EDTA (TE) buffer was pipetted onto the membrane and left to incubate for 10 min at 65°C in a dry bath. The column was centrifuged (10,000 rpm) for 30 seconds to collect the DNA. Assay volume for calculations ~ 100 μL.

#### Near-boiling aqueous solutions

Fifty μL of either RNA-free *water* (Qiagen Sciences Inc., Germantown, MD) or *Prepman Ultra* (Applied Biosystems, Foster City, CA) were added to thawed pellets. Samples were then placed in a dry bath at 99°C for 15 minutes, allowed to cool, and centrifuged for 3 min at 13,000 rpm (15,682 × *g*). The supernatant was transferred to a sterile 1.7 mL micro-centrifuge tube. Assay volume for calculations ~ 50 μL.

#### Trizol max bacterial RNA isolation kit (Invitrogen, Carlsbad, CA)

One mL of *Trizol reagent* was mixed with the thawed bacterial pellet and the sample was incubated at room temperature for 5 minutes at which point 200 μL of chloroform was added. Tubes were vigorously mixed by manual shaking for 10–15 seconds and left at room temperature for 3 min. Samples were centrifuged at 11,000 × *g* (10,888 rpm) for 15 minutes at 6°C, after which the clear upper phase was carefully removed and discarded. Three hundred μL of EtOH was then added and tubes were inverted several times to mix contents. After mixing, samples were kept at room temperature for 3 min then centrifuged (2,000 × *g*) for 5 min at 6°C. The supernatant was carefully removed and pellets were washed with 1 mL 0.1 M sodium citrate in 10% EtOH followed by a 30 minute incubation at room temperature. Samples were again centrifuged (2,000 × *g*) for 5 minutes at 6°C and another wash with the sodium citrate solution, incubation, and centrifugation were repeated. After centrifugation, the supernatant was discarded and the pellets were left to air dry for approximately 45 minutes after which 300 μL of an 8 mM NaOH solution was applied. Lastly, 10 μL of 1 M 4-(2-hydroxyethyl)-1-piperazineethanesulfonic acid (HEPES) buffer was added (pH ~ 7). Assay volume for calculations ~ 300 μL.

#### DNEasy blood & tissue kit (Qiagen Sciences Inc, Germantown, MD)

Two hundred μL of PBS was added to each pellet and 20 μL Proteinase K (solution provided with kit; concentration not reported) were added along with 200 μL Buffer AL and briefly vortexed. Samples were incubated at 56°C in a dry bath for 10 minutes and 200 μL of EtOH was added and mixed. This suspension was transferred onto a filter column and centrifuged for 1 min at 8,000 rpm (*i.e.*, 5,939 × *g*). The collection tube was discarded and replaced, after which 500 μL Buffer AW1 was added, centrifuged at 8,000 rpm for 1 min and collection tube was again discarded. Five hundred μL of AW2 was added to the column and centrifuged for 3 min at 13,200 rpm (*i.e.*, 16,168 × *g*). The filter column was then placed into a clean 1.7 mL centrifuge tube and 200 μL Buffer AE was pipetted onto the center of the membrane. The column was left to sit for 1 minute before centrifuging at 8,000 rpm for 1 minute at which point the DNA solution was collected. Assay volume for calculations ~ 200 μL.

#### “Labiase” enzyme (Sigma-Aldrich, St. Louis, MO)

A 10 mL solution of labiase (5 mg mL^-1^) [14] was made and 300 μL was then added to each bacterial cell pellet and mixed with the pipette. Samples were incubated at 37°C for 3 hours after which samples were subjected to dry bath at 99°C for 15 minutes and then spun down for 3 min at 13,200 rpm whereupon the supernatant was collected. Assay volume for calculations ~ 300 μL. These results have not been included in the tables due to extremely poor results (little change in *C*_*∂j*_ with each *j*^th^ dilution).

#### Genscript BacReady (GenScript Corporation, Piscataway, NJ)

One hundred μL of *Genscript* reagent solution was added to each bacterial pellet. Samples were incubated at room temperature for 4 hours. Because the solutions were turbid after incubation, another 100 μL reagent was added and gently mixed by pipette and incubated overnight at 4°C. Assay volume for calculations ~ 200 μL.

#### Agencourt Genfind v2 (Beckman Coulter, Indianapolis, IN)

A 100 mg mL^-1^ solution of both RNaseA and lysozyme were made but all other solutions used were provided. To each pellet, 400 μL of lysis buffer, 9 μL of a Proteinase K solution (96 mg mL^-1^), 1 μL of a RNaseA solution, and 12 μL of a lysozyme solution were added. Cells were lysed for 10 minutes at 37°C at which point 633 μL of a binding buffer containing magnetic beads (1.5 × sample volume) was pipetted into the samples and left to incubate for 5 minutes at room temperature. The beads were collected on a Dynal MPC-S magnet for 15 min and the supernatant was discarded. The magnetic beads were washed with 1.6 mL of Wash Buffer 1 with gentle re-suspension (pipette), collected again on the magnet, and the supernatant was discarded; repeat this wash step. Subsequently, 1 mL of Wash Buffer 2 was added; this was was performed 2×. Once the second wash was completed, 500 μL of pure water was added to the beads and re-suspended. A two minute incubation at room temperature followed, and beads were magnetically isolated for 5 minutes. The supernatant was then carefully transferred to a sterile 1.7 mL centrifuge tube. Assay volume for calculations ~ 500 μL.

#### QuickExtract bacterial DNA kit (Epicentre Biotechnologies, Madison, WI)

**Figure 1:**
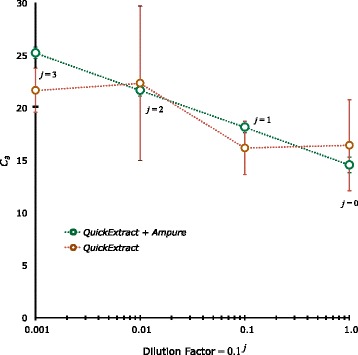
**Semi-Log plot of** ***C***_**∂*j***_ **(** ***QuickExtract*** **±** ***Ampure*** **bead clean-up) as a function of dilution.** Each data point is a mean of 3 replicates ± *s*. These data demonstrate the poor performance of the *QuickExtract* protocol when used without subsequent DNA clean-up (*Ampure* beads).

To each bacterial pellet, 100 μL of *QuickExtract* Bacterial DNA Extraction Solution and 1 μL of lysozyme solution (Epicentre proprietary solution, concentration not provided by manufacturer) was added and tubes were inverted for mixing. Samples were incubated at room temperature for 2 hours. Since all the solutions had not clarified an additional incubation overnight at 4°C was performed. When these samples were tested most had poor *ε*- (0.9 ≥ *ε* ≥ 1.1) and/or ${\rho}_{x\cdot y}^2$ -values (for *x* = *Log*_10_ 0.1^*j*^ and *y* = *C*_∂j_) as well as excessive variability between replicates (Figure 1, red symbols). However, when an *AmPure* DNA clean-up step was used on these samples, the results were far better and are reported herein (Figure 1, green symbols). This step involved adding 72 μL of AmPure magnetic beads (Agencourt Bioscience, Beverly, MA, USA) to 40 μL of the *QuickExtract* solution and mixing thoroughly using a pipette. Magnetic separation of the bead•DNA complex (SPRIplate 96-R magnetic plate) was performed for 5 min and the supernatant was discarded. While on the magnet, beads were washed with 150 μL of 70% EtOH for 30 seconds and the solution discarded. This wash was repeated and after discarding the supernatant, the beads were allowed to air-dry for 30–45 minutes at which point 40 μL of PCR water was added to re-suspend the beads. The beads were separated magnetically for 5 min and 30 μL of the solution was carefully removed. Assay volume for calculations ~ 100 μL.

#### High pure PCR template prep kit (Roche Diagnostics, Indianapolis, IN)

Two hundred μL of PBS was added to the bacterial pellets and re-suspended whereupon 5 μL of lysozyme (10 mg mL^-1^) was added and left to incubate at 37°C for 15 minutes. After incubation, 200 μL Binding buffer was added along with 40 μL Proteinase K (included in kit) and followed with another incubation step at 70°C for 10 minutes. One hundred μL of isopropanol was added and sample was pipetted onto a filter column and spun at 8,000 × *g* for 1 minute. Collection tubes were discarded, replaced and 500 μL Inhibitor Removal buffer was added to the column and spun down at 8,000 × *g* for 1 minute. The collection tube was again discarded, replaced, and 500 μL of Wash buffer was added and spun down at the same rate. The wash was repeated a second time, making sure to discard the flow-through. The column was centrifuged an extra 15 seconds at high speed to ensure that the membrane was dry, then transferred to a new 1.7 mL centrifuge tube where 200 μL of Elution buffer was pipetted onto the membrane and spun down at 8,000 × *g* for 1 minute to collect DNA. Assay volume for calculations ~ 200 μL.

#### “BeadBug” microtube homogenizer (Benchmark Scientific Inc, Edison, NJ) combined with High Pure

Each bacterial pellet was suspended in 1 mL of PBS and transferred to tubes containing *BeadBug* beads (500 μm diameter) especially made for the *BeadBug* homogenizer. Cells were then mixed with the beads for 2 minutes at 3,000 rpm and tubes were spun down for 3 min at 13,200 rpm. The supernatant was carefully collected and dispersed into five sterile 1.7 mL centrifuge tubes, each containing ~ 200 μL. The High Pure extraction was then followed as outlined above. The DNA solution from all 5 tubes, was then combined for future use. Assay volume for calculations ~ 1000 μL.

#### Omnilyse (Claremont BioSolutions, Upland, CA)

The *Omnilyse* device [9] consists of a disposable 3 mL syringe and an attachment with a mixing chamber as well as inlet/outlet ports. A small motor equipped with a “precision-cut impellor” has been installed in the mixing chamber along with the zirconia/silica beads. Upon activation with the included battery, the motor drives the impellor at a rate ≥ 30,000 rpm when the chamber is filled with both sample and beads thereby generating a high shear force between beads causing disruption of cells caught within this “shear flow”. To each pellet, 500 μL of PCR water was added and the cells were re-suspended. The *Omnilyse* syringe apparatus was assembled according to manufacturer directions and connected to the 6 V *Bat-Pac* battery provided with the kit. Before use, each syringe and chamber containing the beads was washed with 500 μL PCR-grade water, consisting of a total of 6 washes (counting both uptake and output) being performed in ~ 1 min. Once this pre-wash process was complete, the syringe was moved to the bacterial suspension and a small amount was drawn up before the battery was turned on. The remainder of the sample was then carefully run through the chamber a total of 18–20 times within a 2 minute time frame. Assay volume for calculations ~ 500 μL.

## Results and discussion

### Experimental approach

In order to perform quantitative metagenomic analyses of native, Eubacterial populations, an efficient set of genomic DNA extraction protocols is desired. Towards this end we have tested a dozen “next generation” DNA extraction procedures recommended by molecular biologist colleagues, or sampled from recent literature, using a *q*PCR assay which works for any “true” bacterial isolate in terms of obtaining target gene copy number per CFU. From prior experience [1] working with the 16S *r*RNA gene extracted from various organisms, we know that *stnd* and *unk* DNA *q*PCR efficiencies can differ substantially and make target DNA quantitation more error-prone than need be. To address this problem, we developed a *q*PCR protocol and simple algorithm [25] which took into account, and corrected, such *ε*-variation between samples of unknown DNA concentration and their associated standard solutions. In the current work, using this approach, we thrice-cultured various test organisms in a randomized complete block design [27], used a drop-plate method [33] to estimate the total CFU mL^-1^ for each biological replicate (or block), numerous 1 mL sub-samples from each of the three cultures per isolate (12× Gram-negative, 6× Gram-positive) were selected, solids (*i.e.*, cells) were centrifuged out, and the pellets were frozen (-20°C) to preserve them for eventual testing as well as assist in breaking, or softening, the cell envelopes [18] with a concomitant diminishment of cell viability [35]. These frozen concentrates of cells were then used for testing commercially available protocols, discussed below. For many of our test organisms, the number of copies of the 16S *r*RNA gene per genome (typically 5–8) is known [36] or can be surmised from knowledge about genetically related types: *e.g.*, using *Listeria* spp. for *Bt* [37,38]. Also, normalization of all gene copy number results to CFUs provides us with a relative measure of the closeness to truly quantitative extraction of genomic DNA.

### Survey of 12 bacterial cell lysis and extraction protocols with *Bt*, *Ss*, and *Ec*

#### DNA extraction results from gram-positive organisms

Table 1 displays *δ*-normalized 16S *r*DNA copy number data (Broc PA/PB primer set) for all extraction protocols performed on 3 biological replicates (blocks) of *Bt*. The reported means have been sorted from lowest to highest and those with a different letter are taken to be “significantly” different at the *P* = 0.05 level. For these *Bt* extractions alone, the *SE* ($\sqrt{EMS\div n}=24.4$) [28] was sizable because of the large variation (0.322 ± 0.0497 to 934 ± 19.9 16S *r*DNA copies CFU^-1^) in the quantity of genomic DNA being extracted from this Gram-positive organism and is testimony to the difficulty in efficiently disrupting such murein-laden cell walls. The *HSD* multiple range test is also quite rigorous (*e.g.*, no significant difference between 0.322 ± 0.0497 [*FastID*] and 22.0 ± 3.57 [*BeadBug*] 16S *r*DNA copies CFU^−1^) due to the relatively large *q*_*P*_ value which, in turn, is due to the sizeable number of comparisons (*m* = 11; the labiase treatment was not included due to poor *q*PCR behavior). Of these eleven DNA extraction methods, 7 were statistically not much better than the extraction of DNA with hot (99°C) RNA-free water (0.655 ± 0.415 copies CFU^-1^). From our experience such a low efficacy in DNA extraction is more expected than not inasmuch as DNA extraction biases, which result in low [*T*]_*j*=0_, can be caused by many problems such as chromosomal shearing and, if pure DNA is required (as when we generate standard concentrations of 16S *r*DNA), loses can occur associated with purification schemes such as *AmPure* magnetic beads (was not obvious in our usage, however: Figure 1). However, the observed large CFU-normalized gene copy number seen using the *Agencourt* procedure (141 ± 48.8 copies CFU^-1^) was not particularly surprising because *Bt* is known to form long (10–20 cells CFU^-1^), linear chains of rod-shaped cells [38].

**Table 1:** **Colony forming unit-normalized 16S** ***r*** **DNA copy number for various extraction protocols associated with three biological replicates of** ***Brochothrix thermosphacta***

	**Brochothrix copies 16S** ***r*** **RNA “gene” per CFU**					
**Extraction methods**	**Culture 1**	**Culture 2**	**Culture 3**	**Mean**	**Stdev**	
Fast ID	0.265	0.353	0.349	0.322	0.0497	*a*
Boiling Water	0.222	1.05	0.693	0.655	0.415	*a*
Trizol	0.484	1.74	0.643	0.956	0.684	*a*
DNEasy	0.602	1.72	1.28	1.20	0.561	*a*
PrepMan	0.916	2.67	2.04	1.88	0.889	*a*
Genscript	8.67	10.9	8.72	9.43	1.27	*a*
BeadBug	18.7	21.6	25.8	22.0	3.57	*ab*
Agencourt	143	189	91.4	141	48.8	*bc*
QuikExtract (Ampure)	329	73.6	248	217	131	*c*
High Pure	596	544	579	573	26.9	*d*
Omnilyse	918	956	927	934	19.9	*e*
		CFU mL^−1^				
	8.62 × 10^6^	6.92 × 10^6^	7.92 × 10^6^			
	±5.66%	±4.46%	±7.44%			

Any two means reported with different letters are significantly different at the *P* = 5 × 10^-2^ level.

However, the very high copy numbers observed using either the *High Pure* (573 ± 26.9 copies CFU^-1^) or *Omnilyse* (934 ± 19.9 copies CFU^-1^) techniques were unexpected. If *Bt* is similar to its nearest relative (*Listeria* spp.), there should be 6× 16S *r*RNA gene copies per genome [36]. Thus, the *Omnilyse* result implies 934 copies CFU^-1^ ÷ 6 copies genome^-1^ ~ 150 genomes CFU^-1^ which suggests a substantially larger number of cells CFU^-1^ than noted above. For some organisms this relatively large 16S *r*RNA gene copy number could be somewhat over-estimated since CFU count on solid media is typically underestimated. For instance, we have found that the ratio of MPN (liquid media-based) to CFU counting methods (pure cultures) for *Campylobacter* spp. = 2.58 ± 0.909 [5]. However, CFU mortality on solid media is problematic for organisms with only ~ 1 or 2 cells CFU^-1^. To achieve a 100% extraction efficiency (*EE*; see Definitions Section, above) with an observation of 934 copies of the 16S *r*RNA gene CFU^-1^ one could reasonably assume: 6 copies genome^-1^, > 100 cells CFU^-1^, no “correction” for growth losses on solid media because of the large CFU size, and 1–2, or more [39], genomes per cell since, during log-phase, some chromosomes can have several “replication bubbles”.

**Figure 2:**
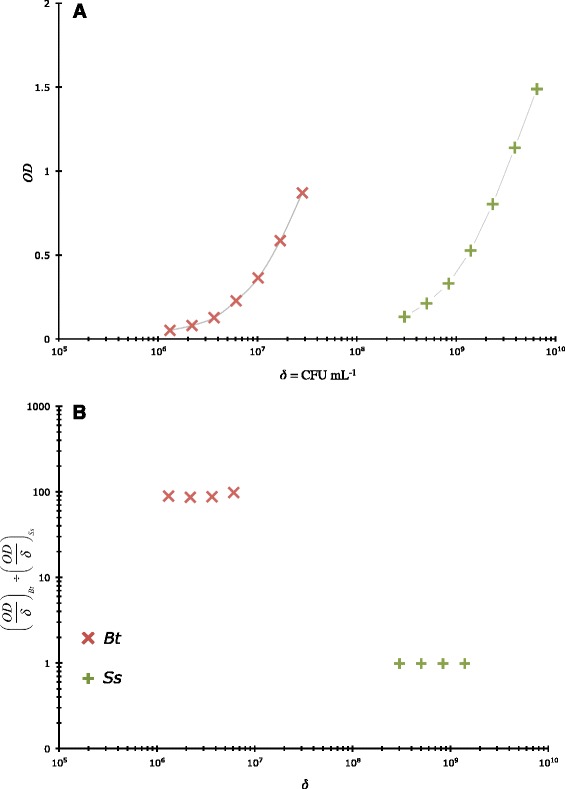
**Estimation of the relative size of colony forming units. A)** Semi-Log plot of optical density at 600 nm as a function of bacterial (× = *Bt*; + = *Ss*) concentration (*δ* = CFU mL^−1^). We have used the semi-Log format in this figure only in order to show all data on the same scale. All results are linear (for OD ≤ 0.5, ${\rho}_{x\cdot y}^2$ = 0.999 [*t*_*ρ*_ = 54.7, *P* = 1.35 × 10^−5^] and 0.997 [*t*_*P*_ = 25.8, *P* = 1.28 × 10^−4^] for *Bt* and *Ss*, respectively) on a non-Log scale. **B)** The ratio of *Bt δ*-normalized OD (~0.5 or less) at 600 nm to this same variable associated with *Ss* plotted with *δ*.

To determine if *Bt* can have more than 100 cells CFU^-1^, we developed an optical measure of CFU size based upon the principle that turbidity of a relatively dilute solution (*e.g.*, OD ≤ 1) of suspended particles is proportional to the number of particles per volume × the projected area per particle. Thus, the turbidity, as measured by the optical density at ~ 600 nm (OD), for bacterial cells of similar size and shape, is directly proportional to something related to number of particles & size of those particles. By normalizing the OD of cell cultures to their cell density (δ) one factors out the particles per volume term. Upon calculating the ratio (OD/δ)_*Bt*_ ÷ (OD/δ)_*Ss*_ one achieves a measure of the number of *Bt* cells CFU^-1^*relative to* the *Ss* standard’s presumed number of cells CFU^-1^ (between 1 and 2 cells CFU^-1^). One such set of data are shown in Figure 2b (OD ≤ 0.5) and indicate that *Bt* can have ~ 100 cells CFU^-1^. Replicating this experiment 6 times more we observed an average (OD/δ)_*Bt*_ ÷ (OD/δ)_*Ss*_ = 128 ± 66.1 (ranging from 76.6 to 269, *n* = 7) for gently mixed cultures (using a 5 mL pipette). Performing the same analyses, but vortexing for 30 sec prior to making dilutions, we observed average (OD/δ)_*Bt*_ ÷ (OD/δ)_*Ss*_ = 85.7 ± 59.9 (ranging from 39.5 to 208, *n* = 7) whereupon these two mixing treatments were significantly different at the *P* = 0.0168 level (“=FDIST(*F*, *m*-1 = 1, (*m*-1)*(*n*-1) = 6)”; *Log*-transformed data). In a different set of comparable experiments, a similar result was obtained by diluting colonies of each organism (*Bt* and *Ss*) in LB broth until an OD of 1 was achieved at which point, upon vortexing, they were 6 × 6 drop-plated plated, grown overnight, and enumerated. Under these conditions we found that the ratio of *Ss* colony counts to *Bt* counts (both for an OD ~ 1) was 151 ± 47.1 (*n* = 7). We contend that the extreme difference (2-log *δ*-separation between *Bt* and *Ss* OD data) in observed *δ* between these two rod-shaped, and similar-sized, organisms at the same OD (Figure 2a) and *can only* be related to differences in CFU dimension. All these results support the concept that *Bt* can have a large number of cells per CFU relative to *Ss* and that the *Omnilyse* technique of cell lysis provided close-to-quantitative genomic DNA extraction from this tough-walled [9], Gram-positive organism. To achieve a complete agreement of the CFU-normalized OD results (average of the three above means = $\overline{x}\pm {s}_{\overline{x}}=121\pm 12.5$) with the CFU-normalized 16S *r*DNA copy data (*i.e.*, 934 ± 19.9 copies CFU^-1^) we merely had to assume a bulk average of 1.28 genomes per cell [39] × 6 gene copies per genome × 121 cells CFU^-1^.

#### DNA extraction results from gram-negative organisms

Tables 2 and 3 display CFU-normalized 16S *r*RNA gene copy number data (Shig 2A/2B primer set) for all extraction protocols (*m* = 11) using *Ss* and *Ec* as target organisms. As with the *Bt* extractions (Table 1), these results demonstrated that the *Omnilyse* (15.1 ± 3.35 copies CFU^−1^ for *Ss*; 26.9 ± 3.39 copies CFU^−1^ for *Ec*), *Agencourt* (20.8 ± 2.68 copies CFU^-1^ for *Ss*; 16.0 ± 5.35 copies CFU^-1^ for *Ec*) and *High Pure* (8.33 ± 1.21 copies CFU^-1^ for *Ss*; 26.0 ± 1.31 copies CFU^-1^ for *Ec*) genomic DNA extraction procedures were consistently the most efficient. It is interesting to note, however, that the *High Pure* + *BeadBug* combined *Ss* extraction treatment displayed 2.07 ± 0.0451 copies CFU^-1^ which is ~ 25% of the *High Pure* alone. Something similar was also observed for *Ec*’s *High Pure* + *BeadBug* treatment (7.31 ± 0.429 copies CFU^-1^ or ~ 28% of the *High Pure* alone). These possible evidences of excessive genomic DNA shearing were exacerbated when the *BeadBug*-based protocol was used on *Bt* (Table 1: 22.0 ± 3.57 copies CFU^-1^ or only ~ 4% of the *High Pure* alone). The *Trizol* (9.44 ± 0.748 copies CFU^-1^ for *Ss*) and *Fast ID* (16.7 ± 3.61 copies CFU^-1^ for *Ec*) methods were relatively effective but for only one each of the 3 tested organisms. It is also curious that the *QuickExtract* (with *AmPure* DNA clean-up) procedure for the Gram-negative organisms was one of the worst (1.23 ± 0.399 copies CFU^−1^ for *Ss*; 3.62 ± 2.46 copies CFU^−1^ for *Ec*) but fairly efficient, albeit variable, for our Gram-positive test organism (Table 1: 217 ± 131 copies CFU^-1^; 289 ± 57.3 with culture #2 removed). Of course, without the *AmPure* purification procedure, the *QuickExtract q*PCR behavior from all three isolates was extremely poor: *e.g.*, 0.8 > *ε*_*unk*_ > 1.2, *ε*_*unk*_ highly variable, and, for *Bt*, *C*_*∂j*_ was not very linear with respect to *Log*_10_[0.1^*j*^] (Figure 1, red symbols). We believe such poor *q*PCR results are an indicator of enzyme perturbation [26] by inhibitory substances in the extract because they were completely reversed upon *AmPure* clean-up whereupon 0.913 < *ε*_*unk*_ < 1.03 as previously demonstrated in Figure 1 (green symbols). In order to observe an 100% *EE* associated with the 20.8 *Ss* 16S *r*DNA copies CFU^-1^ (*Agencourt*) observation, one could judiciously propose: 7 copies genome^-1^ [36], 1.5 genomes cell^-1^ [39], 1.5 cells CFU^-1^ and a 1.32 correction for growth losses on solid media (76% plating efficiency). Similarly, for an 100% *EE* associated with an average of 26.9 *Ec* 16S *r*DNA copies CFU^-1^ (*Omnilyse*) necessitates assuming: 7 copies genome^-1^, 1.5 genomes cell^-1^, 1.5 cells CFU^-1^ and a 1.7 correction (59% plating efficiency) for growth losses on solid media. These plating efficiency correction terms seem unreasonably high (low efficiency) so it is probable that the number of genomes cell^-1^ should be made higher (between 1.5 and 3). Backing up the larger genomes cell^-1^ value is the fact that actively dividing bacteria can contain 2 or more copies of complete, or partially-replicated, chromosomes per cell [39]. Of course, the greatest unknown variables in these calculations is plating efficiency-related correction factor for CFU losses on solid media and the number of genomes per cell. Since the above *EE* calculations seem reasonable and, in fact, vary toward the high end, it is probable that the consistently best extraction procedures (Omnilyse, Agencourt and/or High Pure) are very efficient relative to standard genomic DNA protocols (*PrepMan Ultra* and DNEasy).

**Table 2:** **Colony forming unit-normalized 16S** ***r*** **DNA copy number for various extraction protocols associated with three biological replicates of** ***Shigella sonnei***

	***Shigella*** **copies 16S** ***r*** **RNA “gene” per CFU**					
**Extraction methods**	**Culture 1**	**Culture 2**	**Culture 3**	**Mean**	**Stdev**	
Quick Extract (AmPure)	1.29	0.808	1.60	1.23	0.399	*a*
Fast ID	1.26	1.06	1.82	1.38	0.394	*a*
BeadBug	2.03	2.12	2.07	2.07	0.0451	*a*
PrepMan Ultra	2.62	2.30	3.99	2.97	0.898	*ab*
DNeasy	2.42	2.96	3.63	3.00	0.606	*ab*
Boiling Water	4.70	2.21	3.49	3.47	1.25	*ab*
Genscript	2.22	2.88	9.88	4.99	4.24	*abc*
High Pure	6.96	8.74	9.28	8.33	1.21	*bc*
Trizol	9.03	8.98	10.3	9.44	0.748	*cd*
OmniLyse	18.7	14.4	12.1	15.1	3.35	*de*
Agencourt	18.4	23.7	20.4	20.8	2.68	*e*
		CFU mL^−1^				
	3.87 × 10^8^	3.67 × 10^8^	4.18 × 10^8^			
	±16.7%	±5.35%	±0.940%			

Any two means reported with different letters are significantly different at the *P* = 5 × 10^-2^ level.

**Table 3:** **Colony forming unit-normalized 16S** ***r*** **DNA copy number for various extraction protocols associated with three biological replicates of** ***Escherichia coli*** **O79**

	***E. coli*** **copies 16S** ***r*** **RNA “gene” per CFU**					
**Extraction methods**	**Culture 1**	**Culture 2**	**Culture 3**	**Mean**	**Stdev**	
DNEasy	2.98	2.55	4.62	3.38	1.09	*a*
QuikExtrax (AmPure)	2.07	2.34	6.46	3.62	2.46	*a*
BeadBug	7.43	6.83	7.66	7.31	0.429	*ab*
Trizol	7.07	13.9	3.77	8.25	5.17	*abc*
Boiling Water	11.2	10.0	7.35	9.52	1.97	*abc*
PrepMan Ultra	10.7	14.2	10.5	11.8	2.08	*abc*
Genscript	12.7	12.0	18.9	14.5	3.80	*bc*
Agencourt	11.5	14.5	21.9	16.0	5.35	*bc*
Fast ID	12.5	18.6	18.9	16.7	3.61	*c*
High Pure	25.6	25.0	27.5	26.0	1.31	*d*
OmniLyse	28.6	23.0	29.1	26.9	3.39	*d*
		CFU mL^−1^				
	5.61 × 10^8^	6.04 × 10^8^	6.13 × 10^8^			
	±8.60%	±27.8%	±12.4%			

Any two means reported with different letters are significantly different at the *P* = 5 × 10^-2^ level.

Because the CFU-normalized DNA yields for *Bt* were often more than 50-fold greater than the equivalent for either *Ss* or *Ec*, normal analysis of covariance is unreasonable. Alternatively, however, when the extraction methods for *Bt* were assigned a numerical value from 1 = *Fast ID* to 11 = *Omnilyse* (Table 1 extraction method order: 1, 2, ⋯, 11) and used with either *Ss*’s (Table 2 extraction methods top to bottom: 9, 1, 7, 5, 4, 2, 6, 10, 3, 11, 8) or *Ec*’s (Table 3 extraction methods top to bottom: 4, 9, 7, 3, 2, 5, 6, 8, 1, 10, 11) CFU-normalized DNA yields, one can test the level of correlation between these organisms’ DNA yield covariation with the *Bt*-based method’s order. Neither *Ss*- (*ρ*_*Bt⋅Ss*_ = 0.464 [*t*_*ρ*_ = 1.66, *P* = 0.128]) nor *Ec*-based 16S *r*DNA yields (*ρ*_*Bt⋅Ss*_ = 0.462 [*t*_*ρ*_ = 1.65, *P* = 0.131]) associated with the *Bt*-based numerical treatments gave a “significant” correlation. A similar correlation can be made with the *Ss*-based method order (Table 2) and correlated with *Ec* DNA yields (*ρ*_*Ss⋅Ec*_ = 0.520 [*t*_*ρ*_ = 1.93, *P* = 8.29 × 10^-2^]) indicating that there is a greater correlation, but still not statistically significant, between these two Gram-negative species from the standpoint of DNA yields. In other words, the various extraction protocols do not correlate well between species in terms of total genomic DNA yield.

### Survey of *High Pure*, *Agencourt*, and *Omnilyse* genomic DNA extraction methods used with other foodborne Eubacteria (5 Gram-positive, 10 Gram-negative)

#### DNA extraction results from additional gram-positive organisms

Table 4 displays δ-normalized 16S *r*DNA copy number data (16S FU/16S RU primer set) for the 3 consistently most efficient extraction protocols (*High Pure*, *Agencourt*, and *Omnilyse*) from Tables 1–3 using various Gram-positive Eubacteria as target organisms. For 3 of the tested organisms, the *Omnilyse* device/process gave significantly greater yields of 16S *r*DNA: *C. maltaromaticum* lysis resulted in 7.03 ± 0.746 copies CFU^-1^ which was nearly 6-fold greater than either *High Pure* or *Agencourt*; *E. faecalis* provided 10.9 ± 1.26 copies CFU^-1^ which was more than 3-fold greater than the other two methods; lastly, we obtained 42.6 ± 11.6 copies CFU^-1^ from *S. aureus* which was 87-fold greater than either *High Pure* or *Agencourt*. Of the remaining 2 test isolates, *Omnilyse* was statistically equivalent to the other two extraction methods. The isolates *L. lactis* and *S. aureus* have been reported to have 5–6 copies per genome of 16S *r*DNA [36]. Therefore, assuming 6 copies per genome, 1 genome per cell, and 100% extraction efficiency the *L. lactis* and *S. aureus* results argue for 1 and 7 cells CFU^-1^, respectively. The relatively high number for *S. aureus* is probably reasonable since it has at least several cells CFU^-1^ which, under a microscope, can appear as “grape-like” clusters. (All the isolates discussed herein had been thoroughly vortexed before plating and therefore the relative number of cells CFU^-1^ could be smaller than those shown in photomicrographs.) All the *other* Gram-positive isolates in Table 4 have an unknown number of copies of 16S *r*DNA CFU^-1^ but since these are likely to be ≤ 6, we could argue that the *Omnilyse* procedure is nearly quantitative for these isolates as well.

**Table 4:** **Colony forming unit-normalized 16S** ***r*** **DNA copy number for the best extraction protocols (Tables** **1** **,** **2** **and** **3** **) associated with three biological replicates of various Gram-positive Eubacteria**

		**Copies 16S** ***r*** **RNA “gene” per CFU**					
**Gram Pos isolates**	**Extraction method**	**Culture 1**	**Culture 2**	**Culture 3**	**Mean**	**Stdev**	
*Carnobacterium maltaromaticum*	High Pure	0.711	0.679	1.34	0.909	0.374	*a*
	Agencourt	1.56	1.24	2.07	1.62	0.419	*a*
	OmniLyse	6.20	7.65	7.23	7.03	0.746	*b*
			CFU mL^−1^				
		5.63 × 10^8^	5.34 × 10^8^	3.89 × 10^8^			
		±11.1%	±22.5%	±37.6%			
*Enterococcus faecalis*	High Pure	3.05	2.14	2.76	2.65	0.465	*a*
	Agencourt	3.28	3.75	4.12	3.72	0.421	*a*
	OmniLyse	10.5	9.88	12.3	10.9	1.26	*b*
			CFU mL^−1^				
		7.01 × 10^8^	6.32 × 10^8^	5.90 × 10^8^			
		±0.240%	±7.72%	±1.71%			
*Lactococcus lcatis*	High Pure	4.19	3.11	2.74	3.35	0.753	*ab*
	Agencourt	1.15	1.48	1.44	1.36	0.180	*a*
	OmniLyse	4.58	8.28	5.47	6.11	1.93	*b*
			CFU mL^−1^				
		7.58 × 10^8^	7.55 × 10^8^	8.01 × 10^8^			
		±9.55%	±12.5%	±9.99%			
*Staphylococcus aureus*	High Pure	0.307	0.328	0.349	0.328	0.0210	*a*
	Agencourt	0.568	0.707	0.649	0.641	0.0698	*a*
	OmniLyse	30.3	44.2	53.3	42.6	11.6	*b*
			CFU mL^−1^				
		5.54 × 10^8^	4.58 × 10^8^	4.96 × 10^8^			
		±7.44%	±1.66%	±14.8%			
*Streptococcus pneumoniae*	High Pure	9.95	8.55	7.14	8.55	1.41	*a*
	Agencourt	8.77	9.39	8.62	8.93	0.408	*a*
	OmniLyse	7.90	7.42	6.15	7.16	*0.904*	*a*
			CFU mL^−1^				
		3.24 × 10^8^	3.60 × 10^8^	3.92 × 10^8^			
		±14.6%	±0.937%	±8.37%			

Any two means reported with different letters are significantly different at the P = 0.05 level. These statistical comparisons were made within isolate only.

#### DNA extraction results from additional gram-negative organisms

Tables 5 and 6 exhibit CFU-normalized 16S *r*RNA gene copy number data (16S FU/16S RU primer set) for the 3 reliably best extraction protocols from Tables 1–3 using various Gram-negative Eubacteria as target organisms. Table 5 provides information on the efficiency of genomic DNA extraction from *Aeromonas salmonicida*, *Acinetobacter lwoffii*, *Citrobacter freundii*, *Hafnia alvei*, and *Kluyvera ascorbata*. Using these 5 organisms, *Omnilyse* was the clear statistical winner especially with respect to *A. lwoffii* (101 ± 30.3 [or 83.7 ± 3.54 if culture #2 is removed] copies CFU^-1^ which is more than 20-fold greater than either *High Pure* or *Agencourt*) and *C. freundii* (20.8 ± 3.96 copies CFU^-1^, more than 75-fold greater than the other techniques). Genomic DNA extracted from both *K. ascorbata* (13.2 ± 2.55 copies CFU^-1^) and *H. alvei* (9.75 ± 2.68 copies CFU^-1^) were also statistically better (on average > 2-fold) using the *Omnilyse* method. *A. salmonicida* showed a statistically equivalent extractability of all 3 test methods (ranging from 11.1 ± 0.115 to 17.2 ± 4.09 copies CFU^-1^). Of the 5 organisms in Table 5, we only know the number of 16S *r*DNA copies (6–7) per genome for *A. lwoffii* which therefore implies at least an average of 12 cells CFU^-1^. An internet search for this organism does provide some photomicrographs; one example showed the number of cells CFU^-1^ ranging from 1 or 2 to over 40: $\overline{x}$ ± *s* = 18.5 ± 14.4 (*n* = 12, randomly chosen from a pool of 26); however, some of the cell clustering in photomicrographs is probably due to artifacts associated with sample preparation. Table 6 provides data on the relative efficiency of genomic DNA extraction from *Pantoea agglomerans*, *Pseudomonas oleovorans*, *Rahnella aquatilis*, *Salmonella* Typhi, and *Serratia proteamaculans*. Of these 5 organisms, *Omnilyse* was the statistical front-runner only with respect to *R. aquatilis* (13.8 ± 2.64 copies CFU^-1^ which is a little < 2-fold to > 3-fold greater than either *Agencourt* or *High Pure*, respectively). *Omnilyse* was statistically equivalent to the best of the other methods with *P. agglomerans*, *P. oleovorans*, and *S.* Typhi. Of all the organisms tested, only *S. proteamaculans* showed a relatively poor *apparent* extraction efficiency with the *Omnilyse* procedure (1.18 ± 0.788 copies CFU^-1^ [or 1.60 ± 0.396 if culture #1 is ignored]): assuming 7 copies genome^-1^ [36] × 1 genome cell^-1^ × 1 cell CFU^-1^ ~ 7 copies of the 16S *r*RNA gene CFU^-1^, the *Omnilyse* technique was only ~ 17-23% efficient and the *Agencourt* procedure is about 83% efficient. Using the same assumptions for *S*. Typhi (*e.g.*, 11 copies of 16S *r*DNA CFU^-1^) we estimate > 80% *EE* for *Agencourt* and ~ 60% for *Omnilyse*. It is interesting, therefore, that the Gram-positive isolates give what appears to be near-quantitative genomic DNA isolation using *Omnilyse* but this was not the case for all of the Gram-negative Eubacteria. Because of this latter observation, we hypothesize that some genomic DNA shearing may be occurring for some isolates when using the *Omnilyse* procedure.

**Table 5:** **Colony forming unit-normalized 16S** ***r*** **DNA copy number for the best extraction protocols (Tables** 1 **,** 2 **and** 3 **) associated with three biological replicates of various Gram-negative Eubacteria (A—K)**

		**Copies 16S** ***r*** **RNA “gene” per CFU**					
**Gram Neg isolates**	**Extraction method**	**Culture 1**	**Culture 2**	**Culture 3**	**Mean**	**Stdev**	
*Aeromonas salmonicida*	High Pure	11.0	11.2	11.0	11.1	0.115	*a*
	Agencourt	18.4	12.6	20.5	17.2	4.09	*a*
	OmniLyse	11.8	6.73	15.3	11.3	4.31	*a*
			CFU mL^−1^				
		8.96 × 10^8^	9.82 × 10^8^	7.21 × 10^8^			
		±12.7%	±16.4%	±10.4%			
*Acinetobacter lwofii*	High Pure	4.51	3.09	2.55	3.38	1.01	*a*
	Agencourt	2.97	3.34	2.17	2.83	0.598	*a*
	OmniLyse	86.2	136	81.2	101	30.3	*b*
			CFU mL^−1^				
		1.68 × 10^8^	1.17 × 10^8^	1.59 × 10^8^			
		±10.1%	±0.432%	±16.4%			
*Citrobacter freundii*	High Pure	0.180	0.241	0.251	0.224	0.0384	*a*
	Agencourt	0.300	0.292	0.244	0.279	0.0303	*a*
	OmniLyse	25.1	20.0	17.3	20.8	3.96	*b*
			CFU mL^−1^				
		6.42 × 10^8^	6.26 × 10^8^	6.39 × 10^8^			
		±7.61%	±9.55%	±6.99%			
*Hafnia alvei*	High Pure	3.36	3.58	2.28	3.07	0.696	*a*
	Agencourt	2.57	5.97	4.96	4.50	1.75	*a*
	OmniLyse	12.8	7.78	8.68	9.75	2.68	*b*
			CFU mL^−1^				
		1.62 × 10^9^	1.85 × 10^9^	1.66 × 10^9^			
		±15.9%	±27.5%	±25.5%			
*Kluyvera ascobata*	High Pure	5.68	4.20	3.34	4.41	1.18	*a*
	Agencourt	8.38	10.9	7.37	8.88	1.82	*b*
	OmniLyse	14.0	15.2	10.3	13.2	2.55	*c*
			CFU mL^−1^				
		1.12 × 10^9^	9.24 × 10^8^	1.24 × 10^9^			
		±17.0%	±6.65%	±16.1%			

Means reported with different lettters are significantly different at the P = 0.05 level. These statistical comparisons were made within isolate only.

**Table 6:** **Colony forming unit-normalized 16S** ***r*** **DNA copy number for the best extraction protocols (Tables** **1** **,** **2** **and** **3** **) associated with three biological replicates of various Gram-negative Eubacteria (P—S)**

			**Copies 16S** ***r*** **RNA “gene” per CFU**				
**Gram Neg isolates**	**Extraction method**	**Culture 1**	**Culture 2**	**Culture 3**	**Mean**	**Stdev**	
*Pantoea agglomerans*	High Pure	15.7	10.8	9.97	12.2	3.10	*a*
	Agencourt	13.2	16.2	15.2	14.9	1.53	*ab*
	OmniLyse	20.8	23.3	29.8	24.6	4.65	*b*
			CFU mL^−1^				
		5.04 × 10^8^	5.03 × 10^8^	4.09 × 10^8^			
		±12.4%	±12.6%	±10.9%			
*Pseudomonas oleovorans*	High Pure (TSB)	38.6	30.4	24.9	31.3	6.89	*a*
	Agencourt (TSB)	36.7	42.3	48.9	42.6	6.11	*ab*
	OmniLyse (TSB)	69.9	52.4	58.1	60.1	8.93	*ab*
	OmniLyse (LB)	73.0	120	205	133	66.9	*b*
			CFU mL^−1^ (TSB)				
		5.21 × 10^7^	4.62 × 10^7^	5.17 × 10^7^			
		±7.10%	±0.729%	±7.82%			
			(LB)				
		4.89 × 10^7^	2.70 × 10^7^	1.89 × 10^7^			
		±17.1%	±4.36%	±25.8%			
*Rahnella aquatilis*	High Pure	5.15	5.43	6.28	5.62	0.588	*a*
	Agencourt	7.83	8.57	7.05	7.82	0.760	*b*
	OmniLyse	16.7	13.3	11.5	13.8	2.64	*c*
			CFU mL^−1^				
		6.04 × 10^8^	7.07 × 10^8^	8.13 × 10^8^			
		±16.0%	±13.5%	±16.1%			
*Salmonella Typhi*	High Pure	3.17	3.81	4.69	3.89	0.763	*a*
	Agencourt	10.7	8.10	8.48	9.09	1.40	*a*
	OmniLyse	7.50	5.71	6.39	6.53	0.904	*ab*
			CFU mL^−1^				
		8.98 × 10^8^	7.70 × 10^8^	7.13 × 10^8^			
		±0.750%	±3.72%	±2.60%			
*Serratia preteamaculans*	High Pure	3.24	3.35	6.57	4.39	1.89	*b*
	Agencourt	5.59	5.34	6.57	5.83	0.650	*b*
	OmniLyse	0.325	1.88	1.32	1.18	0.788	*a*
			CFU mL^−1^				
		1.33 × 10^9^	1.19 × 10^9^	1.19 × 10^9^			
		±12.8%	±11.7%	±13.4%			

Any two means reported with different letters are significantly different at the P = 0.05 level. These statistical comparisons were made within isolate only.

## Conclusions

In this work we have evaluated a dozen commercial bacterial genomic DNA extraction methodologies using 3 biological replicates each of *Bt* (Gram-positive), *Ss* (Gram-negative), and *Ec* (Gram-negative). We utilized real time *q*PCR quantitation and two specific sets of primers (one for *Bt* and one other against *Ss* and *Ec*) associated with 16S *r*RNA gene to determine the number of copies per CFU by comparing the 4 dilutions of unknown target DNA extracts using *q*PCR in conjunction with 6 dilutions of standards for each primer set. Dilutions of the unknown extract were made in order to determine if any polymerase chain reaction inhibition was apparent since this would result in an evident non-linear relationship between *C*_∂*j*_ and *Log*_10_[0.1 ^*j*^] and poor *ε*_*unk*_ values (0.9 > *ε*_*unk*_ > 1.1). We observed such an inhibition in only two extraction procedures (*QuickExtract* and *Labiase*). The *QuickExtract* inhibition was overcome (Figure 1) by purifying the extracted genomic DNA using *AmPure* magnetic beads with only minor loss of DNA. Based upon statistical analyses of all our results, we determined that the *Agencourt Genfind v2* (Beckman Coulter), *High Pure PCR Template Prep Kit* (Roche Diagnostics), and *Omnilyse* (Claremont BioSolutions) methods provided the greatest consistent yield of genomic DNA (Tables 1, 2 and 3). Assuming 6-7× 16S *r*RNA gene copies per genome, between 1 and 3 genomes per cell (mid-log phase) and 100 - 200 (*Bt*) or 1–2 cells CFU^-1^ (*Ss* and *Ec*) and a correction for the diminution of CFU survival on solid media, we project that the quantitative extraction of genomic DNA from these isolates should produce ~ 1,000 16S *r*DNA copies CFU^-1^ for *Bt* and 22 to 32 copies CFU^-1^ for either *Ss* or *Ec*. The large number of cells CFU^-1^ implied by the *Bt*-*Omnilyse* results were supported by CFU-normalized OD data (100 to 122 cells CFU^-1^). Taking this into account, the *Omnilyse* procedure appeared to provide near-quantitative extraction of genomic DNA for many of these isolates. These three consistently best-performing methods (*Agencourt*, *High Pure*, *Omnilyse*) were assessed (Tables 4, 5 and 6) using 5 additional Gram-positive isolates and 9 Gram-negative species using a set “universal” 16S *r*DNA primers. The best overall method was found to be *Omnilyse* inasmuch as 72% of the isolates tested gave the greatest recovery with this procedure. For 17% of these isolates, *Omnilyse* was statistically equivalent to the best method (*Agencourt*) for these particular organisms. In 20% of the isolates tested (*S. pneumoniae*, *A. salmonicida*, and *S.* Typhi), the *Omnilyse* extraction procedure provided less genomic DNA than (but not statistically significant) the best method. However, one of the Gram-negative isolates (*S. proteamaculans*; < 2 copies CFU^-1^) showed the *Omnilyse* method was not as efficient as the other methods which might argue that this technique is damaging the DNA in some species.

## Additional files

Additional file 1:
**Ss copies 16S rDNA per CFU.**
Additional file 2:
**Analysis of Variance and Other Statistical Expressions.**
