# Supplementary material for: The near-quantitative sampling of genomic DNA from various food-borne Eubacteria
Source: BMC Microbiol. 2014 Dec 31;14:326. doi: 10.1186/s12866-014-0326-z (PMC4302497; doi:10.1186/s12866-014-0326-z)
Supplement: Additional file 2: — Analysis of Variance and Other Statistical Expressions. [file 12866_2014_326_MOESM2_ESM.docx]

Supplemental Materials:

**Analysis of Variance and Other Statistical Expressions**

= ; is an example of “dot notation” used to simplify/clarify equations involving multiple summation operators [27] whereupon the dot in the subscript indicates that the summations is occurring across the subscript where the dot occurs; Excel formulations follow within “ ⋯ ” and all Excel data arrays were made up of *k* rows × *ℓ* columns

“=SUM($x_{1, 1}:x_{m n}$)”

=

“=SUM($x_{k, 1}:x_{k n}$)”

=

“=SUM()”

SS = Sum of squares

Total SS = ; the Excel equations within “{⋯}” represent an array function input (*e.g.*, Command + Enter [Mac] or Control + Shift + Enter [PC]); for instance, the command “{=SUM(($x_{1, 1}:x_{m n}$)^2)”, indicates that each in the $x_{1, 1}:x_{m n}$ array is first squared and then summed

“{=SUM(($x_{1, 1}:x_{m n}$)^2)-((SUM($x_{1, 1}:x_{m n}$))^2)/(*m***n*)}”

Block SS =

“{=((SUM(()^2))/*m*)-((SUM($x_{1, 1}:x_{m n}$))^2)/(*m***n*)}”

Treatment SS =

“{=((SUM(()^2))/*n*)-((SUM($x_{1, 1}:x_{m n}$))^2)/(*m***n*)}”

**HSD algorithm**

The “Tukey Multiple Range” (“Honestly Significant Difference”) tests were made more facile by sorting means from low to high and applying to each *k*^th^ treatment (row vectors from *k* = 1 [smallest mean] to *k* = *m* [greatest mean]) the Excel-based relationship

“ =IF(ABS($\bar{x}_{1}-\bar{x}_{ k \geq1}$)<$q_{0.05}$*SQRT(*EMS*/3),"NS","S")”

for every *k* (*i.e.*, if $\left| \bar{x}_{1}-\bar{x}_{2} \right|$ is less than $q_{0.05}$×$\sqrt{EMS\div3}$ then the difference is *not significant* = *NS*, otherwise it is *significant* = *S* ); in the adjacent column of data (*i.e.*, *k^τ^* = 2; τ represents a transposition operation from a row to column format) then

“ =IF(ABS($\bar{x}_{2}-\bar{x}_{ k \geq2}$)<$q_{0.05}$*SQRT(*EMS*/3),"NS","S")”

for every *k* ≥ 2; in the next column (*k^τ^* = 3) then

“ =IF(ABS($\bar{x}_{3}-\bar{x}_{ k \geq3}$)<$q_{0.05}$*SQRT(*EMS*/3),"NS","S")”

for every *k* ≥ 3; this progression is continued until the *m ^τ^*-*th* column is reached whereupon

“ =IF(ABS($\bar{x}_{m}-\bar{x}_{ m}$)<$q_{0.05}$*SQRT(*EMS*/3),"NS","S")”

always gives *NS*. Letters used to symbolize the above-defined “statistically significant” mean differences were easily assigned once the above “S ” or “NS ” relationships are generated as outlined above.
